# The overdose epidemic: a study protocol to determine whether people who use drugs can influence or shape public opinion via mass media

**DOI:** 10.1186/s40352-022-00189-3

**Published:** 2022-07-23

**Authors:** Ehsan Jozaghi, VANDU

**Affiliations:** 1 UBC Faculty of Dentistry, Nobel Biocare Oral Health Centre, 2151 Wesbrook Mall, Vancouver, BC V6T 1Z3 Canada; 2 Vancouver Area Network of Drug Users, 380 E Hastings St, Vancouver, BC V6A 1P4 Canada

**Keywords:** Fentanyl, People who use drugs, Media, Public opinion, VANDU, Epidemic, Criminalization, Marginalization, Newspapers, Magazine, Advertisement, Human rights

## Abstract

**Background:**

We are currently witnessing an ongoing drug overdose death epidemic in many nations linked to the distribution of illegally manufactured potent synthetic opioids. While many health policy makers and researchers have focused on the root causes and possible solutions to the current crisis, there has been little focus on the power of advocacy and community action by people who use drugs (PWUDs). Specifically, there has been no research on the role of PWUDs in engaging and influencing mass media opinion.

**Methods:**

By relying on one of the longest and largest peer-run drug user advocacy groups in the world, the Vancouver Area Network of Drug Users (VANDU), newspaper articles, television reports, and magazines that VANDU or its members have been directly involved in will be identified via two data bases (the Canadian Newsstream & Google News). The news articles and videos related to the health of PWUDs and issues affecting PWUDs from 1997 to the end of 2020 will be analyzed qualitatively using Nvivo software.

**Discussion:**

As our communities are entering another phase of the drug overdose epidemic, acknowledging and partnering with PWUDs could play an integral part in advancing the goals of harm reduction, treatment, and human rights.

## Introduction

Many nations, including Australia, Canada, the United Kingdom, and the United States have shown rapid increases in cases of opioid overdose deaths (Giraudon et al., 2013; Gomes et al., 2014; Roxburgh et al., 2013; Rudd et al., 2016). There are indications that a shift towards a global epidemic of opioid use and misuse is also inevitable, if appropriate preventative and harm reduction measures are not implemented (Degenhardt et al., 2013; Nolan et al., 2018). In fact, in the US, there were nearly 100,000 Americans who died of a drug overdose in 2020, and since the COVID-19 pandemic, overdose death rates have increased 30% compared to the prior year (Cartus et al., 2020; Lambdin et al, 2022). While researchers and policy makers have offered causes and possible solutions to the current opioid epidemic, many jurisdictions have already implemented numerous initiatives aimed at reducing drug overdose deaths.

One such initiative that has been implemented in many regions is the public health/awareness campaigns about the potency of synthetic opioids through media and paid public advertisement (Kerr et al., 2013; Hedberg et al., 2018; Soukup-Baljak et al., 2015). Mass media, such as television, radio, newspapers, and magazines can be used effectively to reach large proportions of populations and change public opinion. In fact, recent public health campaigns, most notably the effects of tobacco use, have shown the success of mass media in changing social norms, attitudes, and behaviors related to addictive behavior (Wakefield et al., 2010). Similar results have been shown in other health areas, such as the National High Blood Pressure Education Program and the National Cholesterol Education Program, where large scale national campaigns helped to reduce both blood pressure and blood cholesterol in the United States (Roccella, 2002).

While mass media campaigns through paid advertisement could be successfully implemented to change attitudes, norms, perceptions, and eventually behavior, there has been little focus on the power of advocacy and community action by marginalized groups to accomplish similar outcomes. This is especially true for people who use drugs (PWUDs) who not only face numerous barriers in accessing health care, but have historically faced discrimination, stigmatization, and criminalization for their chronic relapsing medical condition (Lunze et al., 2015; Kiriazova et al., 2017; Rivera et al., 2014; Jozaghi, 2013; Thomson et al., 2017).

However, recent research that evaluates the social and scientific impact of North America’s longest and largest drug user advocacy group, the Vancouver Area Network of Drug Users (VANDU), has demonstrated that PWUDs are capable of collectively advocating for health and wellbeing of marginalized groups (Jozaghi et al., 2018). Furthermore, prior ethnographic studies have noted that PWUDs through VANDU and its affiliate drug user lead groups have the capacity to not only advocate and give voice to the most marginalized members of society, but they have the capability to transform the discriminatory rhetoric around the war on drugs (Boyd et al., 2017; Brown et al., 2018; Goodman et al., 2017; Jozaghi, 2014a, 2014b, 2015; Jozaghi & Reid, 2014; Jozaghi et al., 2020; Maynard et al., 2021; Olding et al., 2018).

### Explanation and justification of method

Therefore, if PWUDs have the power to lead, collaborate, and facilitate ground breaking research while advocating for the members of their community, then the question of whether PWUDs can inform and engage public opinion via mass media is topical. This is especially pivotal, because there has been no research thus far to evaluate such power of engagement and community action via mass media by PWUDs.

However, the previous work evaluating the effects of framing language, repetitive stories and agenda-setting signify how public perception and solutions to a problem (Happer et al., 2013; Scheufele et al., 2007) can be affected by media coverage. Generally, the predominate U.S. news media portrayal of the opioid crisis has shown why media and public opinion matter, and how PWUDs may have been left out of the discussion and participation. For example, most of the US media coverage of the opioid crisis has been focused on framing the PWUDs’ issues as a criminal justice rather than of public health (Wild et al., 2019). In fact, a study that considered the 15th year period of the major U.S. newspapers (e.g., the fourth highest circulating newspapers), more than 60% of opioid crises news stories reported on prosecution, enforcement, and harsher penalties rather than relative treatment and/or harm reduction (e.g., 4% only focused on harm reduction) (McGinty et al., 2016).

Although qualitative content analysis of news items can almost always be descriptive in nature; the qualitative content analysis of the Canadian news media coverage of opioid crises have been examined previously. Such studies have shown the capability of the media in shaping public discourse on developments of harm reduction by selectively reporting on harm reduction and naloxone, leading to increased public support for these strategies compared to other alternatives (Wild et al., 2019).

For example, a recent comprehensive reporting of a major Canadian Newspaper reported significant increase in the coverage of opioid reported issues since 2010 with “peaks in coverage coincided with major public health or policy developments, indicating that opioids and their misuse became and have remained a priority issue in the eyes of the public over the years” (Quan et al., 2020, p. 8). Finally, Quan et al. reported that “the language used to describe people who use drugs has shifted to less stigmatizing language in recent years” (p. 8).

However, a comprehensive portrayal of the PWUDs in engaging newspaper reporting is limited and poorly understood mainly due to stigma attached to drug users as sources of knowledge and expertise. Although this is the first time that media engagement of drug user’s advocacy effort has been proposed to be evaluated via a content analysis, McLean (2017) has previously examined the media framings of overdose via qualitative analysis of news items. McLean (2017) reported that PWUDs’ addiction is viewed through the lens of discourse of disposal and the impropriety of punitive responses to drug misuse. The methodological approach of this protocol is inspired by Mclean (2017) approach where the data extraction and analysis (e.g., data base searches, NVivo software usage and data analysis) are initiated by coding and framing of “noticeable, meaningful, or memorable” information.

### Objective

Therefore, this study protocol will examine the following goal:

By identifying, analyzing and reporting on news articles, videos, and magazines that have been written, reported, and influenced by VANDU’s membership, this article aims to show the level of engagement of marginalized groups with mass media.

## Methods

### Data source

In order to effectively conduct the qualitative content analysis of news items, this protocol will rely on a news data base: 1) the ProQuest Canadian Newsstream (formerly the Canadian Newsstand Complete) and 2) Google News. The Canadian Newsstream via ProQuest web interface was chosen because it provides not only the largest newspaper, video, and magazine article data bases in Canada (full text items of nearly 300 unique newspapers and news organizations), but the timeline for many of the articles goes beyond many of the other archive bases (some items date back to the 1970s) (Rosenbloom, 2018; Rosenbloom, 2019). Furthermore, the ProQuest Canadian Newsstream is able to provide access to other major Canadian news sources (e.g., the Globe & Mail, CBC News, and Maclean's) while at the same time allowing many regional media sources to be easily attainable (e.g., the Hamilton Spectator, the Medicine Hat News, and the Vancouver Courier) (Rosenbloom, 2018).

Google News was chosen as the second data source because it is a free based news portal that is simplified based on public use, rather than solely for academic purposes (Weaver et al., 2008). More than a decade ago, it was estimated that more than 9 million people access their news from Google news each month (Weaver et al., 2008); now it is estimated that 280 million people access their daily news from the noted platform (Franek, 2022). At the same time, Google News has a broader appeal in today’s society, as more and more people are relying on social media such as YouTube and Google Chrome products to access news and up-to-date information via the clustering of news information on topics of interest (Banerjee et al., 2007). This is especially important in the context of this research, as the main objective relates to mass media’s role in shaping public opinion.

In addition, one of the main advantages of Google News over all other archival data bases, such as ProQuest, Factiva, JSTOR, Periodicals Archive Online, or LexisNexis, is its ability to provide not only text items, but other graphics (e.g., photos) and background information (e.g., headline size and story placement) that are often eliminated before a story is archived in traditional data bases (Weaver et al., 2008). Finally, Google news is able to capture local news or smaller non-print articles (e.g., VICE, the Georgia Straight, the Tyee, the North Shore News, and rabble.ca) that are often missed by other data bases, such as in this case, the ProQuest Canadian Newsstream.

### Data collection/Sampling

The data pertaining to this study protocol related to openly accessible news items available to the public via Google or the membership/subscriber-based academic portal of Canadian Newsstream; therefore, no ethics approval was needed from a university or institution. The search query for both Google News and Canadian Newsstream involved the phrase, “Vancouver Area Network of Drug Users”. No restriction on the publication date of news items was considered for both data bases, because some web interfaces and search engines have no details by which they order their results (Haddaway et al., 2015).

The inclusion criteria for news items (e.g., newspapers, magazines, and videos) will be based on the following criteria: 1) items written or spoken in the English language; 2) items that are related directly to VANDU’s recent activism, work, or action; 3) items that involve members of VANDU (e.g., action, comments, or activism). The news items that met the initial inclusion criteria will be further reviewed in full before being added for qualitative analysis. The English language was chosen because most of VANDU’s activism happen in the British Columbia province and the Canadian Newstream includes the English content.

As shown on Table 1, the preliminary data collection, from the Canadian Newsstream, indicates that 1,196 articles have been shaped by VANDU membership since 1997.

**Table 1:** The Canadian Newsstream articles shaped by VANDU membership

Time Ranges Start	Time Range End	Number of Results
1997–01-01	1997–12-31	4
1998–01-01	1998–12-31	11
1999–01-01	1999–12-31	15
2000–01-01	2000–12-31	49
2001–01-01	2001–12-31	32
2002–01-01	2002–12-31	100
2003–01-01	2003–12-31	106
2004–01-01	2004–12-31	66
2005–01-01	2005–12-31	69
2006–01-01	2006–12-31	55
2007–01-01	2007–12-31	33
2008–01-01	2008–12-31	84
2009–01-01	2009–12-31	43
2010–01-01	2010–12-31	56
2011–01-01	2011–12-31	50
2012–01-01	2012–12-31	42
2013–01-01	2013–12-31	45
2014–01-01	2014–12-31	36
2015–01-01	2015–12-31	23
2016–01-01	2016–12-31	68
2017–01-01	2017–12-31	55
2018–01-01	2018–12-31	44
2019–01-01	2019–12-31	47
2020–01-01	2020–12-31	63

During the full review, each news item’s geographic area will be divided via an excel worksheet into local (e.g., British Columbia (B.C.)), national (e.g., Canada), and International categories. Moreover, the data range for each article will also be noted in the excel worksheet.

## Material/Data handling

In addition to Microsoft Excel which will be used for quantitative analysis linking geography and important dates to the data set, this study protocol also relies on Nvivo software (version 12) for the qualitative analysis. Version 12 of Nvivo software (QSR International Pty Ltd., 2018) has many new features that not only allow for greater exploration and visualization into the qualitative analysis (e.g., coding videos, photos, maps, interviews, pdf files), but also allows for easy cross tabulation of data and information exchange via other software. Many of these features will help in the analysis of news articles and better visualization of imported items.

### Data analysis

The data analysis of news items that have passed the first and second review will be imported into Nvivo for further qualitative analysis. Members of VANU will be employed as community researchers to perform some of the reviews and coding. The duplicate news items that have been picked-up by other news outlet but are identical to the original report will be omitted by the two reviewers.

The initial qualitative analysis will involve open coding, where, based on the review of the whole news item (e.g., the title and news content), each article will be placed into its own category. Placing a whole news item in a coding category in Nvivo is achieved based on the inductive principles of grounded theory, whereas during the data collection, there will already be interplay between coding and data review (Charmaz, 2006; Haddaway et al., 2015; Maher et al., 2018; Suddaby, 2006). Line-by-line coding (Charmaz, 2006) will also be employed to analyze each sentence when descriptive labels will be linked to each sentence or paragraph. The coding will be reviewed for accuracy by the VANDU members and senior author where the clustered themes based on similarities will be assessed. Despite some variation in individual coding, general themes regarding the news passages will be established.

In effect, during the final review and subsequent uploading of the news items, there could be ‘constant simultaneous comparison between items that belong to specific coding categories and whether a new coding category needs to be constructed (Suddaby, 2006). Codes will be developed in an iterative process, as emergent news items and themes will be identified in accordance with grounded theory principles (Strauss & Corbin, 1990). In fact, the emergent themes will be constantly compared with established codes to observe similarities and differences across categories.

To provide linkage and examples from the new inductive, open, and emergent coding (Wilson et al., 2018) Nvivo software will be used once again for a deductive approach in coding the content of news items (Elo et al., 2008; Hsieh et al., 2005; Vaismoradi et al., 2013). During this stage of analysis, latent content analysis will be employed, where the focus is not only on the interpretation of the content (Holsti, 1969), but the main objective is to display the intended usage of the words and phrases (Morse, & Field, 1995; Catanzaro, 1988). Therefore, by relying on Nvivo’s word frequency query, the most frequent words and phrases will be identified. This quantification of the most frequent words or phrases is an attempt to contextualize the text of the news items, rather than infer meaning (Hsieh et al., 2005). In other words, the goal is to find the underlying meaning of the news articles via better analysis of news text.

### Rigor

Later on, the most frequent words and phrases will be used within the context of the previous inductive codes to form new templates and code guides as a means of sorting the text within each news item (Crabtree, & Miller, 1999). The theme identified through this deductive process will help provide further interpretation in terms of latent meanings (Fereday et al., 2006). Since validity is an important concept in research regardless of the methods used, this research also presents findings with quotes from each news item to present the context for each theme and as a standalone representation of the theme. Furthermore, the coding theme and quotes representing each theme will be reported to the VANDU board directors for comments and feedback so validity and reliability could be established based on the oral history of the membership. During data analysis and coding, the research team will meet regularly to compare notes and discuss new themes that could have emerged and consequently refine the framework for coding to fully account for all the theme and topics. Through comparing the narrative data based on the social justice framework, the PWUDs’ experiences of engagement with media and news outlet will be assessed via VANDU membership’s firsthand experiences. VANDU feedback on these themes not only enhances the rigor of our findings but helps in establishing validity in our qualitative findings.

## Results

Not Applicable because it is a study protocol and data has not been collected, analyzed and reported.

## Discussion

While previous research related to the effectiveness of public opinion and public campaign has been conducted through surveys (Kerr et al., 2013; Hedberg et al., 2018; Soukup-Baljak et al., 2015), it is important to note that survey research has historically underestimated the effectiveness of an intervention because of the stigmatized nature of drug use (Shield et al., 2012; Zhao et al., 2009). Therefore, one of the strengths of this research is its unique ability to report on the largest and longest running drug user advocacy group in North America and its impact on public opinion, through mass media engagements. Qualitative findings could indicate the detailed type of activism and community action conducted by the members of the drug user community in forms of interviews, demonstrations, and news reports.

It is important to note that social stigma, where society via mass media endorses stereotypes or negative feelings against an already marginalized group (Livingston et al., 2012), manifests itself at a personal level. As the current opioid epidemic has demonstrated, stigmatization can also manifests at a structural level. Therefore, this research could demonstrate that drug users and their allies could have a profound influence on mass media engagement. In effect, positive stories initiated by peer-led advocacy groups, such as VANDU via mass media engagement could have the power to slowly change the stigma that target PWUDs.

It is also important to note that while previous research has clearly demonstrated the activism and community action of VANDU since the late 1990s, such as numerous demonstrations, night time syringe distributions, injection support teams, and the implementation of unsanctioned supervised injection facilities (SIF), unsanctioned inhalation facilities, scientific research, and education (Jozaghi, 2014a, 2014b, 2015; Jozaghi & Reid, 2014; Jozaghi et al., 2018), VANDU’s mass media engagement as the longest run drug user organization has not been reported in any previous study. One of the shortcomings of this study protocol was related on the reliance of English only speaking News outlets. Also, relying on newspaper accounts can also limit the exact topic of interest might be covered. Therefore, investigating a mixed method approach via surveys research combined with qualitative work may provide the greatest insights, rather than a single methodological approach.

## Conclusion

Therefore, since the formation of VANDU as a response to the public health declaration of blood borne infections (e.g., HIV and hepatitis C cases) and drug overdose cases (e.g., 300 cases per year) in the late 1990s by the Vancouver/Richmond Health Board (Kerr et al., 2006), there has been numerous mass media engagements by VANDU’s memberships. This study protocol attempts to show how VANDU’s past mass media engagements via qualitative content analysis have had impacts on mass media. Perhaps VANDU engagement with the media over time has the potential to show PWUDs’ advocacy efforts and project a shift in public opinion on drug policy. As the overdose crisis linked to synthetic opioids continues, VANDU’s mass media engagements study could demonstrate the level of advocacy by PWUDs for harm reduction (e.g., SIFs), de-criminalization, and human rights.

## Data Availability

Available via request from the corresponding author.

## Abbreviations

B.C.: British Columbia
PWUDs: People who use drugs
SIF: Supervised injection facility
VANDU: Vancouver Area Network of Drug Users
